# Lung and diaphragm ultrasound in noninvasive respiratory support: A real tool or fashion?

**DOI:** 10.5578/tt.20239902

**Published:** 2023-03-10

**Authors:** D. Di Costanzo, M. Mazza, P. Ruggeri, J.B. Blanco, B. Mina, G. Fiorentino, B. Lazovic, G. Scaramuzzo, A. Esquinas

**Affiliations:** 1 Unit of Pulmonology and Respiratory Pathophysiology, Clinic of Medical Sciences, Aorn Sant’Anna and San Sebastiano Hospital, Caserta, Italy; 2 Unit of Pulmonology, Department of Biomedical and Dental Sciences, Messina University Faculty of Medicine, Messina, Italy; 3 Intensive Care Unit, Hospital Garcia de Orta, Almada, Portugal; 4 Clinic of Pulmonary and Critical Care, Lenox Hill Hospital, New York, United States; 5 Clinic of Pathophysiology and Respiratory Rehabilitation, Monaldi Hospital Azienda Ospedaliera Dei Colli, Napoli, Italy; 6 Pulmonogy Ward, University Clinical Hospital Center Zemun, Belgrade, Serbia; 7 Department of Translational Medicine, Ferrara University Faculty of Medicine, Ferrara, Italy; 8 Intensive Care Unit, Hospital Morales Meseguer, Murcia, Spain

**Keywords:** ultrasonography, noninvasive ventilation, respiratory insufficiency

## Abstract

**ABSTRACT:**

Lung and diaphragm ultrasound in noninvasive respiratory support: A real tool or fashion?

**Introduction:**

Over the past few years, there has been an increase in lung and diaphragm ultrasound applications as a tool to evaluate the outcomes and settings of noninvasive respiratory supports. However, actual clinical practices in this field are yet to be known. The aim of this study was to investigate the current clinical utilization of ultrasound for noninvasive respiratory supports on an international level.

**Materials and Methods:**

The study employed an online survey consisting of 32 items, which was sent via email to intensivists, pulmonologists, emergency medicine physicians, and other specialists with expertise in using ultrasound and/or noninvasive respiratory supports.

**Results:**

We collected 52 questionnaires. The ultrasound study of diaphragm dysfunction was well-known by the majority of respondents (57.7%). Diaphragm performance was used as a weaning failure predictor (48.5%), as a predictor of noninvasive ventilation failure (38.5%) and as a tool for the ventilator settings adjustment (30.8%). In patients with acute respiratory failure, 48.1% used ultrasound to assess the damaged lung area to set up ventilatory parameters, 34.6% to monitor it after noninvasive ventilation application, and 32.7% to match it with the ventilatory settings for adjustment purposes. When administering high flow nasal cannula - oxygen therapy, 42.3% of participants used ultrasound to evaluate lung involvement and assess flow parameters.

**Conclusion:**

Lung and diaphragm ultrasound is an established clinical practice to evaluate noninvasive respiratory supports outcomes and settings. Further studies are needed to evaluate the educational aspects to increase confidence and indications for its use.

## Abstract (tr)

**ÖZ:**

Noninvaziv solunum desteğinde akciğer ve diyafram ultrasonu: Gerçek bir araç mı yoksa moda mı?

**Giriş:**

Son birkaç yılda, noninvaziv solunum desteklerinin sonuçlarını ve ayarlarını değerlendirmek için bir araç olarak akciğer ve diyafram ultrason uygulamalarında bir artış olmuştur. Bununla birlikte, bu alandaki gerçek klinik uygulamalar henüz bilinmemektedir. Bu çalışmanın amacı, uluslararası düzeyde non-invaziv solunum desteklerini uygularken ultrasonun klinik kullanımındaki mevcut durumunu araştırmaktır.

**Materyal ve Metod:**

Çalışmada, yoğun bakım, göğüs hastalıkları ve acil tıp hekimlerine, ultrason ve/veya noninvaziv ventilasyon deneyimi olan diğer uzmanlara 32 madde içeren çevrimiçi bir anket e-posta yoluyla gönderildi.

**Bulgular:**

Elli iki anket sonucu kaydedildi. Diyafram disfonksiyonunun ultrason değerlendirmesi, katılımcıların çoğunluğu (%57,7) tarafından iyi biliniyordu. Diyafram performansını, weaning başarısızlığının öngörücüsü olarak (%48,5)’i, noninvaziv ventilasyon başarısızlığının öngörücüsü olarak (%38,5) ve ventilatör ayarlarının ayarlanması için (%30,8) olarak kullanıldığı görüldü. Akut solunum yetmezliği olan hastaların %48,1’inde ventilasyon parametrelerini ayarlamak için, %34,6’sında noninvaziv ventilasyon uygulamasını takiben, %32,7’sinde ise mekanik ventilatör parametrelerini ayarlama amacıyla ultrason kullanılmaktaydı. Yüksek akışlı nazal kanül-oksijen tedavisi sırasında, katılımcıların %42,3’ü akım parametrelerini değerlendirmek amacıyla ultrason kullanmaktaydı.

**Sonuç:**

Akciğer ve diyafram ultrasonu, noninvaziv solunum desteği sonuçlarını ve ayarlarını değerlendirmek için kullanılan bir klinik uygulamadır. Kullanımına yönelik güveni ve endikasyonları arttırmak için eğitimsel yönleri değerlendiren daha ileri çalışmalara ihtiyaç vardır.

## Introduction

Over the past few years, there has been a marked increase in lung and diaphragm ultrasound (LU) use and a growing number of physicians who have integrated it into their daily clinical practice. Of particular interest are the applications of LU as a tool to evaluate the outcomes and settings of noninvasive respiratory supports (NRSs).

The use of lung ultrasound (LU) to identify changes in diaphragm size and function has been extensively studied as a tool to predict successful weaning from mechanical ventilation ( 1 ), as well as to evaluate patient response to noninvasive ventilation (NIV) and NIV failure ( 2 , 3 ). Moreover, an increasing number of studies explored its use to detect patient-ventilator asynchronies (PVA) during NIV ( 4 ).

In addition, lung parenchyma ultrasonography has been identified as a useful tool to assess lung aeration, venous congestion, and lobar/translobar consolidation ( 5 , 6 ). In this context, LU may be helpful in clinical practice to correlate lung aeration and the outcome of NIV in patients with acute respiratory failure (ARF) ( 7 ).

Despite the growing body of evidence, the current clinical practices regarding the use of LU in the application of NRSs and its perceived impact on decision-making are not yet fully understood.

This study aimed to explore the current situation in the clinical use of LU at the international level and to address the issues set out above.

## MATERIALS and METHODS

An online questionnaire including 32 items was created ad hoc by a pool of experts in noninvasive monitoring and NIV. 9/32 items focused on the respondents’ characteristics assessment, 12/32 items on LU diagnostic/monitoring purposes (covering most of the pathologies investigated with LU), 8/32 items on the use of LU related to ARF and NIV (assessing/modifying parameters and patient monitoring, including the aspects concerning diaphragm ultrasound) and 3/32 items to evaluate the use of LU when applying high-flow nasal cannulaoxygen therapy (HFNC-OT). Items concerning the use of LU allowed a binary answer (Yes/No questions).

The survey was sent to intensivists, pulmonologists, emergency medicine physicians, and any other specialists who have experience in the use of LU and/ or NRSs.

The specialists were invited via email to participate in the study and complete the questionnaire using an online platform.

The survey was conducted using the Google Form online application, and no incentives (monetary or non-monetary) were offered to participants. There were no conflicts of interest, and due to the nature of the study, neither informed consent nor approval by a Research Ethics Committee was required. Data collection and treatment were carried out anonymously.

## Results

We collected 52 complete questionnaires. Respondents were from Italy (n= 20/52, 38%), Turkey (n= 8/52, 15%), Mexico (n= 5/52, 9.6%), Argentina (n= 2/52, 3.9%), Greece (n= 2/52, 3.9%), Portugal (n= 2/52, 3.9%), India (n= 2/52, 3.9%), China (n= 2/52, 3.9%), Serbia (n= 2/52, 3.9%), Lebanon (n= 1/52, 2%), Belgium (n= 1/52, 2%), Switzerland (n= 1/52, 2%), USA (n= 1/52, 2%), Egypt (n= 1/52, 2%), Spain (n= 1/52, 2%), and Japan (n= 1/52, 2%).

Respondents were pulmonologists (38.5%), intensivists (36.5%), emergency medicine physicians (9.6%), or other specialists (15.4%). They mainly used conventional cart-based ultrasound (US) (51.8%) and portable US scanners (38.5%), while hand-held US scanners were the least used (9.6%).

In most of the cases, the physicians acquired their ultrasound skills through an accredited course (61.5%) or had learned by performing LU under expert supervision (25%), while a lesser number (13.5%) were self-taught by reading articles, and manuals, or other resources.

Only 7.7% of respondents had less than one year of experience practicing lung ultrasound, 28.8% had 1-3 years of experience, 25% had 3-5 years, 25% had 5-10 years, and 13.5% had more than 10 years of experience.

Regarding the frequency of use of LU in clinical practice, most participants (38.5%) performed LU more than once a week, 28.8% less than once a week, 25% daily, and 7.7% less than once a month.

Regarding the diagnostic and monitoring purposes for which LU was used, 98.1% of physicians used LU for pleural effusion assessment (identification, location, quantification, and drainage orientation), 92.3% to monitor the effectiveness and complications of the pleural effusion drainage, 92.3% for pneumothorax diagnosis (identification, location, quantification, and drainage orientation). Moreover, LU was mostly used for lung consolidation examination (86.5%) and cardiogenic pulmonary edema determination (84.6%). Lastly, 59.6% of respondents used LU in association with echocardiography and venous echography, for the pulmonary embolism analysis and 23.1% for endotracheal tube confirmation after placement of an artificial airway.

Concerning the LU use related to NIV ( Table 1 ), 57.7% of respondents used LU for the diagnosis of diaphragm dysfunction using both diaphragm thickness (DT) and diaphragm excursion (DE). 30.8% of the physicians used DT and/or DE to adjust ventilator parameters (i.e., pressure support), 13.5% coupled DT and/or DE with ventilator curves analysis to detect asynchronies, 38.5% used diaphragm dysfunction as a predictor of NIV failure and 48.5% as a predictor of weaning failure.

In patients with ARF, 38.5% of respondents used LU to evaluate the initial lung involvement as a mortality predictor, and 53.8% to follow up the evolution of lung implication. When NIV was applied, in patients with ARF, 48.1% used LU to assess the damaged lung area to set up ventilatory parameters, 34.6% to monitor it after NIV application, 32.7% to match it with the ventilatory settings (i.e., pressures) for adjustment purposes.

When applying HFNC-OT ( Table 2 ) in patients with ARF, 42.3% of participants used LU also to evaluate the lung involvement and assess HFNC-OT flow, 17.3% to follow up the lung involvement evolution after HFNC-OT application and 21.2% performed LU with HFNC-OT parameters (i.e., flow) adjustment purpose.

Lastly, 53.8% used combined approaches (e.g., LU and echocardiography) to find potential cardiac causes for failure of weaning from NIV.

**Table 1:** Lung ultrasound and noninvasive ventilation

Question	Yes, n(%)	No, n(%)
Do you use LU for the diagnosis of diaphragm performance using diaphragm thickness (DT)?	30 (57.7)	22 (42.3)
Do you use LU for the diagnosis of diaphragm performance using diaphragm excursion (DE)?	30 (57.7)	22 (42.3)
Do you use any of the LU scores to evaluate the lung involvement in patients with respiratory failure and predict mortality?	20 (38.5)	32 (61.5)
Do you use any of the LU scores to follow up on the evolution of lung involvement in patients with respiratory failure?	28 (53.8)	24 (46.2)
Do you use LU to evaluate the lung involvement in patients with respiratory failure and assess the ventilation parameters?	27 (51.9)	25 (48.1)
Do you use any of the LU scores to follow up on the evolution of lung involvement in patients with respiratory failure after NIV application?	18 (34.6)	34 (65.4)
Do you use LU to evaluate lung involvement with ventilator parameters (pressures) adjustment purposes?	17 (32.7)	35 (67.3)
Do you use the analysis of diaphragm function (DT and/or DE) with ventilator parameters (pressure support) adjustment purposes?	16 (30.8)	36 (69.2)
Do you use the analysis of diaphragm function (DT and/or DE) coupled with ventilator curves analysis to detect asynchronies?	7 (13.5)	45 (86.5)
Do you use the analysis of diaphragm function (DT and/or DE) for detecting diaphragm dysfunction as a predictor of NIV failure?	20 (38.5)	32 (61.5)
Do you use the analysis of diaphragm function (DT and/or DE) for detecting diaphragm dysfunction as a predictor of weaning failure?	25 (48.1)	27(51.9)
Do you use combined approaches (e.g., LU and echocardiography) to find potential cardiac causes for failure of weaning from NIV?	28 (53.8)	24 (46.2)

**Table 2:** Lung ultrasound and high flow nasal cannula oxygen therapy (HFNC-OT)

Question	Yes, n(%)	No, n(%)
Do you use LU to evaluate the lung involvement in patients with respiratory failure and assess HFNC-OT flow?	22 (42.3)	30 (57.7)
Do you use any of the LU scores to follow up on the evolution of lung involvement in patients with respiratory failure after HFNC-OT application?	9 (17.3)	43 (82.7)
Do you use LU with HFNC-OT parameters (flow) adjustment purposes?	11 (21.2)	41 (78.8)

## DISCUSSION

The findings of this survey indicate that the use of ultrasound for evaluating the lung and diaphragm is widely adopted in patients receiving noninvasive respiratory support.

The use of ultrasound to identify changes in diaphragm size and function through different indices (i.e., diaphragm excursion, diaphragm thickness, and thickening fraction) has been extensively studied as a feasible, valid, and noninvasive tool to predict successful weaning from mechanical ventilation ( 1 ). Furthermore, the assessment of diaphragmatic dysfunction has been established to evaluate patient response to NIV and predict its failure (2,3). Apart from NIV outcome, there is an increasing interest to explore the use of diaphragmatic ultrasound to detect PVA during NIV ( 4 ). Data collected through our survey showed that the evaluation of diaphragm performance was well-established. More than half of the respondents used LU to examine diaphragm thickness and excursion.

Diaphragm dysfunction was mainly used as a predictor of weaning failure, while its use was less common as a predictor of NIV failure and as a tool for adjusting ventilator settings (i.e., pressure support).

Just a small percentage of respondents coupled the use of DT and/or DE with ventilatory curves analysis to detect asynchronies.

Lung parenchyma ultrasonography has been identified as a helpful tool in the assessment of aeration, congestion, and consolidation. It is considered a valid method of tracking dynamic changes in pulmonary congestion with higher sensitivity and specificity than clinical examination and chest radiography ( 5 , 6 ). In this context, LU has also been incorporated into clinical practice to study the relationship between lung involvement/lung aeration, assessed with US examination, and the outcome of NIV in patients with ARF ( 7 ).

According to our data, in patients with ARF, a small percentage of the physicians used the ultrasound evaluation of initial lung involvement as a mortality predictor, while LU was used mainly for the followup of the evolution of lung implication. When NIV was applied, in patients with ARF, LU was mainly used to assess the damaged lung area to set up ventilatory parameters while a smaller percentage of respondents used LU after NIV application to follow up the lung involvement progression or match it with the ventilatory settings (i.e., pressures) for adjustment purposes.

Of particular interest, in patients with ARF, we found that LU was used to assess lung involvement and assess HFNC-OT flow. In a minor number of cases, LU was used to follow up on the evolution of lung involvement after HFNC-OT application and/or with HFNC-OT parameters (i.e., flow) adjustment purpose.

Despite a growing body of evidence, the actual contribution of ultrasound to NIV response or setting when applying NIV remains unclear. Understanding the use of ultrasound in NIV application and its contribution to NIV response and setting at the international level is essential to identify areas that require further evidence. In this way, researchers will be able to detect potential difficulties in further developing this technique and set up recommendations or training programs to help physicians achieve higher-quality care.

Our study has some limitations: first of all, this is an exploratory study on the use and application of ultrasound monitoring during noninvasive support. The geographical distribution of the responders may provide a generalizable result, although there may be imbalances between different areas. The majority of the participants, 38%, were from one country (Italy). Moreover, a selection bias related to personal interest in the topic cannot be excluded and therefore further studies are needed to confirm our findings.

## CONCLUSION

In conclusion, ultrasound monitoring of the diaphragm and lung is an established clinical practice to evaluate both NIV settings and outcomes. Further studies are needed to evaluate the educational aspects of this practice and determine the need for training initiatives to increase confidence and provide appropriate indications for its use.

## Ethical Committee Approval

The authors declare that due to the nature of this study, neither informed consent nor approval by the research ethics committee was required.

## CONFLICT of INTEREST

The authors declare that they have no conflict of interest.

## AUTHORSHIP CONTRIBUTIONS

Concept/Design: All of authors

Analysis/Interpretation: DDC

Data acqusition: DDC

Writing: DDC

Clinical Revision: All of authors

Final Approval: All of authors
